# Transformative educational leadership as organizational practice: a practice theory perspective on reconfiguring teacher agency and professional culture in schools

**DOI:** 10.3389/fsoc.2026.1847035

**Published:** 2026-08-07

**Authors:** Nur Efendi, Muh Ibnu Sholeh, Nurul Indana, Fahrur Rosikh, Choiruddin, Nur ‘Azah, Sholihan, Imro'atus Solikah, Riyanto

**Affiliations:** 1 Universitas Islam Negeri Sayyid Ali Rahmatullah Tulungagung, Tulungagung, Indonesia; 2 STAI KH Muhammad Ali Shodiq Tulungagung, Tulungagung, Indonesia; 3 IAI Al-Urwatul Wutsqo Jombang, Jombang, Indonesia; 4 Universitas Sunan Drajat Lamongan, Lamongan, Indonesia; 5 Universitas Hasyim Asy'ari Tebuireng Jombang, Jombang, Indonesia; 6 Institut Agama Islam Hasan Jufri Bawean, Gresik, Indonesia

**Keywords:** educational organizations, Global South, labor process, organizational practice, professional agency, professional culture, sociology of work, transformative leadership

## Abstract

Educational leadership has increasingly been understood as a relational and practice-based phenomenon rather than an individual managerial attribute. However, limited research has examined how transformative educational leadership functions as an organizational practice that simultaneously reconfigures teachers' work, professional agency, and professional culture, particularly within Islamic educational institutions in the Global South. This study employed a qualitative multiple-case study design involving three public Islamic senior high schools (Madrasah Aliyah Negeri) in Tulungagung, Indonesia. Data were collected through semi-structured interviews, participant observations, and institutional document analysis involving principals, teachers, and administrative staff. Data were analyzed using thematic analysis and cross-case comparison. The findings reveal that transformative educational leadership operates through everyday organizational practices that restructure teachers' work from individual and routine-based labor into collaborative and reflective professional activity. Leadership practices also expand professional agency through participatory decision-making and foster professional cultures characterized by trust, dialogue, collaboration, and shared responsibility. The findings further indicate the emergence of hybrid professionalism in which teachers derive professional legitimacy simultaneously from organizational accountability, collegial collaboration, and religious-moral responsibility. This study demonstrates that transformative educational leadership functions as an organizational practice that mediates the relationship between institutional structures and professional agency. By reconfiguring teachers' work and professional culture, leadership contributes to organizational adaptability and sustained innovation. The study extends Practice Theory within educational leadership research and contributes to the sociology of professions by demonstrating how transformative leadership mediates professional jurisdiction and fosters hybrid professionalism within Global South educational organizations.

## 1 Introduction

Educational leadership has conventionally been framed as an individual attribute associated with managerial competence, authority, and strategic decision-making within formal organizational hierarchies. Such a perspective emphasizes the role of school leaders as primary agents of change while underestimating the social and organizational processes through which leadership is enacted (Gurr, 2015). Recent developments in organizational sociology offer an alternative lens by conceptualizing leadership as a situated practice emerging from interaction, coordination, and shared meaning within institutional contexts. Leadership, in this sense, is not confined to formal roles but is continuously produced through everyday activities that organize work and shape professional relations (Herepath and Kitchener, 2016; Raelin, 2016).

This shift toward a practice-based understanding aligns with broader transformations in the sociology of work, where attention is directed to how labor is structured, negotiated, and experienced within organizations. In educational settings, teachers' work extends beyond instructional delivery to include collaborative planning, emotional engagement, and participation in institutional governance (Epstein, 2018). These dimensions of work are shaped through leadership practices that define responsibilities, enable coordination, and influence patterns of professional interaction. Empirical research suggests that leadership grounded in distributed and practice-oriented approaches facilitates the reorganization of professional work by enabling collective engagement and shared responsibility (Gronn, 2002). Such an approach highlights the relational and processual nature of leadership in shaping organizational life.

The Indonesian madrasah system provides a particularly relevant context for examining leadership as organizational practice due to its hybrid institutional configuration. Madrasah operate under a dual framework that integrates state-regulated educational policies with Islamic pedagogical values (Almutairi, 2020). This institutional arrangement generates a complex organizational environment where multiple logics coexist and interact. School leaders are required to navigate administrative accountability while sustaining religious and moral commitments embedded in educational practices (Avolio and Yammarino, 2013). The coexistence of bureaucratic and cultural-religious expectations creates conditions in which leadership must function as a mediating practice that aligns diverse institutional demands (Islamic et al., 2023; Samad et al., 2023).

Within this context, professional expectations placed on teachers further illustrate the complexity of organizational dynamics. Teachers in madrasah are expected to perform instructional roles while also embodying moral and community-oriented responsibilities. These expectations contribute to shaping professional agency, understood as the capacity of individuals to act purposefully and influence their work conditions (Priestley et al., 2016). Agency is not an inherent property but emerges through interaction with structural conditions and leadership practices. Research in educational sociology indicates that participatory and dialogic forms of leadership create opportunities for teachers to exercise agency by involving them in decision-making processes and collaborative initiatives (Biesta et al., 2015). Such practices enable teachers to move beyond compliance toward active engagement in organizational development.

From a sociology of professions perspective, these dynamics also raise questions regarding professional jurisdiction, defined as the authority of a profession to define problems, apply expertise, and control work processes (Abbott, 1988). In many Northern professional contexts, professionalization is often associated with increasing autonomy and jurisdictional control over specialized domains of practice. However, the organizational realities of Indonesian madrasahs suggest a different pattern. Teachers operate within overlapping domains of authority shaped by state bureaucracy, school leadership, religious-moral expectations, and collegial professional communities. Professional work is therefore not governed by a single jurisdictional authority but emerges through ongoing negotiation among multiple institutional actors. This context provides an opportunity to reconsider professional jurisdiction as a relational and shared process rather than an exclusive professional monopoly.

At the same time, the presence of bureaucratic governance introduces structural constraints that may limit professional autonomy. Standardized policies, accountability mechanisms, and administrative demands often regulate the scope of teachers' work and restrict opportunities for innovation. These conditions generate tensions between institutional control and professional discretion. Leadership plays a critical role in mediating this relationship by interpreting policy frameworks, negotiating their implementation, and creating spaces for localized adaptation (Ghamrawi, 2023). Studies on school leadership demonstrate that leaders who engage in interpretive and collaborative practices are more effective in aligning institutional requirements with professional needs, thereby fostering adaptive organizational environments (Hsiao and Chang, 2011; Salas and Masluhah, 2024).

Professional culture constitutes another central dimension through which leadership operates as an organizational practice. Culture refers to shared norms, values, and interactional patterns that guide behavior within institutions. It is continuously produced through social interaction rather than being statically embedded in organizational structures. Leadership contributes to the formation and transformation of professional culture by shaping communication practices, facilitating collaboration, and supporting collective learning processes (Wang et al., 2022). Transformative leadership, in particular, is associated with the development of collaborative cultures characterized by trust, openness, and shared responsibility. These cultural shifts influence how teachers engage with their work and with one another, reinforcing patterns of professional interaction that sustain organizational change (Schein, 2010).

Despite the growing body of literature on educational leadership, limited attention has been given to how leadership operates as an organizational practice that simultaneously reconfigures work, agency, and professional culture, particularly within the context of Islamic schooling in the Global South. Existing studies often focus on leadership effectiveness or instructional outcomes without examining the underlying processes through which leadership reshapes organizational dynamics. This gap calls for a sociologically grounded analysis that situates leadership within the interplay of structure, agency, and culture.

In addition to understanding leadership as an organizational practice, this study engages with broader debates in the sociology of professions concerning the nature of professionalism in hybrid educational organizations (Al Ayyubi et al., 2025; Habibulloh et al., 2025). Evetts (2011) distinguishes between occupational professionalism, characterized by collegial authority, trust, professional discretion, and peer regulation, and organizational professionalism, characterized by managerial control, accountability mechanisms, performance indicators, and bureaucratic procedures (Evetts, 2011). While this distinction has been widely applied in educational settings, it may be insufficient for understanding professional work within Global South educational institutions where professional legitimacy is shaped simultaneously by state regulations, community expectations, and religious-moral commitments.

Indonesian madrasahs represent a particularly relevant context for examining this issue because teachers operate within overlapping institutional logics that combine bureaucratic accountability, pedagogical responsibilities, and Islamic ethical obligations. Consequently, professionalism cannot be understood solely as either occupational or organizational. Instead, professional practice emerges through the negotiation of multiple sources of authority and legitimacy. This study therefore explores how transformative educational leadership mediates these tensions and contributes to the emergence of a form of hybrid professionalism that reflects the realities of educational organizations in the Global South.

Beyond conventional transformational leadership approaches that primarily emphasize leader characteristics and organizational performance, transformative educational leadership focuses on changing organizational conditions that enable professional growth, participation, and collective agency. Transformative leadership seeks not merely to improve organizational outcomes but to reshape the social relations through which educational work is organized and experienced. This perspective aligns closely with organizational sociology and Practice Theory, where leadership is understood as a collective accomplishment emerging through interaction rather than individual authority.

Accordingly, this study positions transformative educational leadership as an organizational practice capable of reconfiguring teachers' work, expanding professional agency, and transforming professional culture. By examining leadership through this sociological lens, the study contributes to educational leadership scholarship by shifting attention from leaders as individuals toward leadership as an ongoing organizational process enacted through everyday practices.

While existing studies have extensively examined transformational leadership in relation to school effectiveness, instructional improvement, and organizational performance, relatively little attention has been given to leadership as an organizational practice embedded in everyday work processes. Most educational leadership studies continue to conceptualize leadership as an individual competency or managerial function rather than as a socially situated practice through which work, agency, and culture are continuously produced and reproduced. Drawing on Practice Theory, this study shifts the analytical focus from leaders to leadership practices, emphasizing how organizational actors collectively enact leadership through interaction, coordination, and shared meaning-making. This perspective provides a sociological explanation of how transformative leadership reconfigures teachers' work, professional agency, and professional culture within hybrid educational organizations in the Global South.

## 2 Literature review

Practice Theory provides a useful framework for understanding leadership as a socially situated and collectively enacted phenomenon. Rather than viewing leadership as an attribute possessed by individuals, Practice Theory conceptualizes organizational life as constituted through recurrent practices that organize work, shape social relations, and produce institutional outcomes (Schatzki, 2001; Nicolini, 2012). Practices consist of interconnected activities, shared understandings, norms, and material arrangements that guide action within organizations.

From a practice-theoretical perspective, leadership is not located solely in formal positions but emerges through everyday interactions, routines, and collaborative activities. Leadership is enacted when organizational actors coordinate work, negotiate meaning, solve problems, and construct shared understandings of organizational goals (Raelin, 2016). This approach shifts attention from leaders to leadership practices, highlighting how organizational change is produced through collective action.

In educational organizations, leadership practices influence how teachers organize their work, exercise professional agency, and construct professional culture. Practice Theory therefore offers an appropriate analytical framework for examining how transformative leadership operates as an organizational practice that reconfigures labor processes and professional relationships within schools.

### 2.1 Leadership in organizational sociology

Leadership within organizational sociology is increasingly conceptualized as a distributed and practice-based phenomenon shaped through social interaction rather than formal hierarchy. This perspective challenges traditional views that position leadership as the function of a single authority figure. Leadership emerges through relational processes, routines, and collective engagement that organize work and coordinate action across institutional settings. A practice-based lens highlights how leadership is enacted through everyday activities such as communication, negotiation, and problem-solving, which collectively shape organizational outcomes (Crevani et al., 2010).

This approach aligns with relational theories of leadership that emphasize interaction as the central mechanism through which influence is exercised. Leadership is not located in individuals but in the dynamic relations among actors within organizations. These relations generate patterns of coordination that structure professional work and institutional processes. Empirical research in organizational studies demonstrates that distributed leadership fosters shared responsibility and enhances collective capacity, particularly in knowledge-intensive environments such as schools (Bolden, 2011). Such an understanding situates leadership within broader organizational dynamics, where authority is negotiated and enacted through practice.

In educational contexts, this reconceptualization is significant because schools operate as complex social organizations characterized by interdependent roles and collaborative processes. Leadership practices influence how tasks are distributed, how decisions are made, and how professional relationships are structured. A sociological perspective allows for the examination of leadership as a mechanism that organizes work and shapes institutional culture rather than as a set of individual competencies.

### 2.2 Teachers' work as socially constructed labor

Teachers' work is increasingly recognized as a form of socially constructed labor that extends beyond classroom instruction. It encompasses administrative responsibilities, emotional engagement, and collaborative practices that are shaped by institutional expectations and organizational contexts. This broader understanding reflects shifts in the sociology of work, where labor is viewed as embedded within social relations and organizational structures. Teaching involves not only the transmission of knowledge but also the management of relationships, identities, and institutional demands (Connell, 2020).

The concept of emotional labor is particularly relevant in understanding teachers' work. Teachers are expected to regulate their emotions, maintain supportive relationships with students, and create positive learning environments. These expectations require ongoing emotional engagement that is often invisible yet central to professional practice. Research indicates that emotional labor contributes significantly to the complexity of teaching as a profession, influencing both job satisfaction and professional identity (Miller et al., 2019).

Administrative and collaborative dimensions further illustrate the multifaceted nature of teachers' work. Teachers participate in curriculum planning, institutional evaluation, and professional development activities that require coordination with colleagues and alignment with organizational goals. These activities are shaped by leadership practices that structure opportunities for collaboration and define expectations of professionalism. From this perspective, teachers' work is not fixed but continuously produced through interaction with organizational processes and institutional norms.

Understanding teaching as socially constructed labor provides a foundation for analyzing how leadership practices influence the organization of work within schools. Leadership shapes how tasks are distributed, how responsibilities are negotiated, and how professional roles are defined. This approach highlights the importance of examining work as a dynamic and relational process within educational organizations.

### 2.3 Professional agency and institutional constraints

Professional agency refers to the capacity of individuals to act purposefully, make decisions, and influence their work environment. Within organizational settings, agency is shaped by the interplay between structural conditions and social interaction. It is not an individual attribute but a relational achievement that emerges through engagement with institutional contexts. Organizational structures, leadership practices, and cultural expectations collectively shape the conditions under which agency is exercised.

Institutional structures provide both opportunities and constraints for professional action. Policies, regulations, and accountability systems define the boundaries within which teachers operate. These structures can enable coordinated action while also limiting flexibility and innovation. Research in educational policy highlights how accountability regimes influence teachers' practices by prioritizing measurable outcomes and standardization (Ball, 2003). Such conditions shape the extent to which teachers can exercise discretion in their work.

Leadership practices play a critical role in mediating the relationship between structure and agency. Leadership that emphasizes participation, dialogue, and collaboration creates spaces for teachers to contribute to decision-making processes and organizational development. These practices support the emergence of agency by enabling teachers to interpret policies, adapt practices, and engage in innovation. Studies in organizational learning suggest that agency is strengthened when individuals are involved in collective processes that allow for reflection and shared problem-solving (Goller and Paloniemi, 2017).

Cultural expectations further shape professional agency by influencing norms of behavior and interaction within organizations. Professional culture defines what is considered acceptable, valued, and legitimate in practice. Teachers' actions are guided by these cultural norms, which are continuously negotiated through social interaction. Leadership contributes to shaping these expectations by modeling practices, facilitating communication, and establishing organizational priorities.

An integrated perspective that considers structure, leadership, and culture provides a comprehensive understanding of professional agency in educational settings. Agency emerges through the interaction of these elements, reflecting the dynamic nature of organizational life. This perspective supports the analysis of how leadership practices reconfigure not only work but also the capacity of individuals to act within institutional contexts.

### 2.4 Professional culture and transformative educational leadership

Professional culture refers to the shared values, beliefs, norms, and patterns of interaction that guide professional behavior within organizations (Schein, 2010). In educational settings, professional culture shapes how teachers collaborate, engage in collective learning, and respond to organizational change. Rather than existing as a static organizational characteristic, culture is continuously produced and reproduced through everyday interactions among organizational members.

Transformative educational leadership plays a significant role in shaping professional culture by creating conditions that support collaboration, trust, dialogue, and collective responsibility. Leaders influence culture not only through formal policies but also through the practices they encourage and the interactions they facilitate. Research suggests that schools characterized by collaborative professional cultures demonstrate higher levels of teacher engagement, organizational learning, and innovation (Hargreaves and Fullan, 2015).

From a practice-theoretical perspective, professional culture emerges through repeated organizational practices that reinforce shared understandings and collective commitments. Leadership contributes to this process by organizing opportunities for collaboration, supporting professional dialogue, and enabling collective problem-solving. Consequently, transformative leadership can be understood as a mechanism through which professional culture is continuously negotiated and transformed within educational organizations.

### 2.5 Professionalism in hybrid educational organizations: extending evetts from a global south perspective

The sociology of professions has increasingly questioned the universality of professionalism models developed in Western contexts. Evetts (2011) differentiates between occupational professionalism and organizational professionalism as two dominant modes through which professional work is regulated (Evetts, 2011; Eli Dwi Nabila, 2025; Pervin, 2025). Occupational professionalism emphasizes professional discretion, collegial trust, and peer-based authority, whereas organizational professionalism relies on managerial control, accountability systems, performance monitoring, and formal procedures.

Educational institutions in the Global South frequently operate within institutional environments that do not fit neatly into this dichotomy. In contexts such as Indonesian madrasahs, teachers' professional legitimacy derives not only from professional expertise and organizational accountability but also from religious values, community expectations, and moral responsibilities. Professional authority is therefore distributed across multiple institutional domains rather than located exclusively within either professional communities or organizational structures.

This study adopts the concept of hybrid professionalism to explain how teachers navigate these overlapping expectations. Hybrid professionalism refers to a form of professional practice in which legitimacy is simultaneously derived from state accountability, collegial collaboration, religious responsibility, and community trust. Transformative educational leadership becomes important because it mediates these institutional logics and enables professional work to be coordinated across multiple sources of authority. This perspective contributes to emerging Global South scholarship that seeks to broaden existing theories of professions beyond assumptions developed in Northern contexts.

### 2.6 Professional jurisdiction and leadership mediation in hybrid educational organizations

Abbott (1988) theory of professional jurisdiction explains that professions establish and maintain authority over specific domains of work through claims of specialized expertise, legitimacy, and social recognition. Professional jurisdiction defines which profession has the authority to diagnose problems, make decisions, and regulate professional practice within a particular institutional context (Abbott, 1988). Complementing this perspective, Freidson (2001) argues that professional authority is sustained through professional autonomy, discretionary decision-making, and institutional protection, enabling professionals to exercise independent judgment while maintaining public trust (Freidson, 2001).

Within Global South educational settings, professional arrangements are frequently characterized by institutional plurality and overlapping sources of legitimacy. Teachers' professional work is simultaneously influenced by policy requirements, organizational expectations, cultural norms, religious commitments, and community relationships. Consequently, professional authority cannot be fully understood through traditional models that assume clear professional boundaries and exclusive control over practice (Noordegraaf, 2015).

Transformative educational leadership may therefore be conceptualized as a mechanism of jurisdictional mediation. Rather than simply empowering teachers, leadership coordinates and negotiates competing institutional demands while creating opportunities for professional discretion. This perspective extends conventional assumptions that professionalization necessarily involves increasing autonomy and instead highlights the relational nature of professional authority within hybrid educational organizations (Noordegraaf, 2016).

### 2.7 Theoretical integration and conceptual model

The three strands of literature discussed above—leadership as organizational practice, teachers' work as socially constructed labor, and professional agency under institutional constraints—can be integrated into a unified sociological framework that explains how educational leadership operates as a transformative force within school organizations.

A practice-based understanding of leadership positions it as a relational and distributed process embedded in everyday organizational life. Leadership is enacted through interaction, coordination, and meaning-making that shape how work is organized and experienced (Parkin, 2016). This perspective connects directly with the sociology of work, where labor is understood as socially constructed through institutional arrangements and professional interactions. Teachers' work is continuously negotiated through leadership practices that define responsibilities, enable collaboration, and structure professional expectations (Priestley and Philippou, 2018).

The integration of these perspectives reveals that leadership does not operate in isolation but functions as a mediating mechanism between structure and agency. Organizational structures such as policies, accountability systems, and institutional norms provide the conditions within which teachers perform their work. These structures can both enable and constrain professional action. Leadership practices interpret and translate these structures into everyday practices, shaping how teachers experience their work and exercise agency (Bellibaş et al., 2021).

Professional agency emerges within this interaction as a relational achievement rather than an individual trait. It is shaped by opportunities for participation, dialogue, and collaboration that are facilitated through leadership practices. When leadership is enacted in a participatory and distributed manner, teachers are more likely to engage in decision-making processes and contribute to organizational development. This dynamic supports the transformation of professional culture, where shared norms, trust, and collective responsibility become central to institutional functioning (Hilal et al., 2024).

The concept of transformative educational leadership can be further understood through Bolden (2011) distributed and relational perspective on leadership (Bolden, 2011). Bolden argues that leadership is not concentrated within individual leaders but emerges through networks of interaction among organizational members. Such a perspective resonates strongly with Practice Theory because both approaches conceptualize organizational change as the outcome of collective action rather than individual intervention.

Within educational organizations, transformative leadership functions by enabling participation, fostering shared responsibility, and creating opportunities for collaborative meaning-making. Leadership becomes a practice through which institutional structures are interpreted, negotiated, and adapted to local conditions. Consequently, transformative educational leadership serves as a mediating mechanism connecting organizational structures, professional agency, and professional culture.

This understanding extends conventional leadership theories by emphasizing relational processes over individual traits. Leadership is therefore viewed as an organizational practice capable of transforming not only institutional outcomes but also the social conditions under which professional work is performed.

The integration of work, agency, and culture highlights a recursive relationship. Leadership practices reconfigure teachers' work by redistributing roles and responsibilities. Changes in work structure create new opportunities for agency, enabling teachers to act more autonomously and collaboratively. These changes, in turn, contribute to the development of a professional culture that reinforces collaborative practices and sustained organizational learning. Culture then feeds back into leadership practices by shaping expectations, norms, and patterns of interaction within the organization.

Recent studies in educational leadership emphasize that such transformations are particularly significant in contexts characterized by institutional hybridity and complexity. In settings like madrasah, where bureaucratic governance intersects with religious and cultural expectations, leadership plays a critical role in aligning multiple institutional logics. Transformative leadership practices enable schools to navigate these complexities by fostering adaptive work structures and collaborative professional cultures (Bolden, 2011).

**Figure 1:**
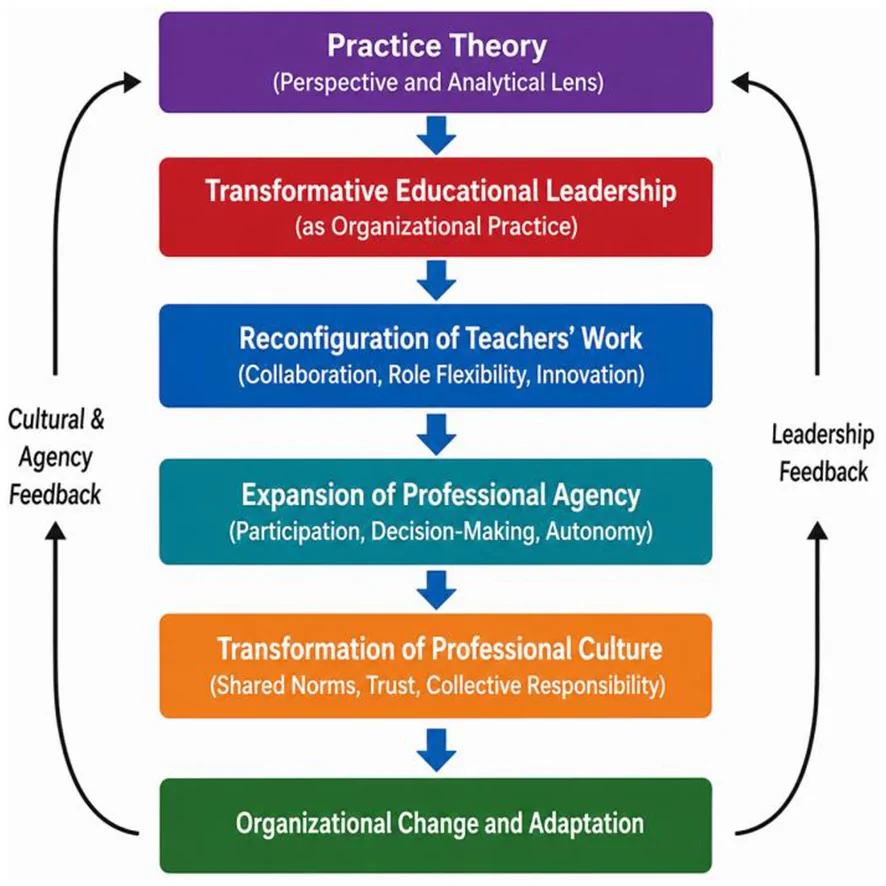
Transformative educational leadership as organizational practice in reconfiguring work, agency, and professional culture.

This integrated framework contributes to the sociology of work and organizations by conceptualizing educational leadership as a practice that actively reorganizes labor processes, redistributes agency, and reshapes institutional culture. It extends existing research by demonstrating how leadership operates across multiple dimensions of organizational life rather than being confined to formal authority or instructional improvement (Figure 1).

## 3 Methodology

### 3.1 Research design

This study adopts a qualitative multiple-case study design to examine how transformative educational leadership operates as an organizational practice in reconfiguring work, agency, and professional culture. A multiple-case approach enables in-depth exploration of complex social phenomena within real-life contexts while allowing analytical comparison across cases (Yin, 2009). This design is particularly appropriate for investigating leadership as a relational and practice-based process embedded in institutional settings.

The study is grounded in an interpretivist paradigm, which emphasizes the meanings constructed by participants through their lived experiences. This perspective allows for an understanding of how leadership practices are enacted, negotiated, and interpreted within school organizations. Qualitative inquiry is suitable for capturing the complexity of organizational life and the social processes that shape professional practices (Creswell and Poth, 2016).

### 3.2 Research sites

The research was conducted in three state Islamic senior high schools (Madrasah Aliyah Negeri) located in Tulungagung, Indonesia. The sites were selected purposively to reflect variation in organizational characteristics and leadership orientations, enabling comparative analysis across different institutional contexts (Table 1).

**Table 1:** Research sites and characteristics.

School	Characteristics	Leadership orientation
**MAN 1 Tulungagung**	High-performing	Transformative
**MAN 2 Tulungagung**	Moderate innovation	Transitional
**MAN 3 Tulungagung**	Developing	Administrative

This variation provides a basis for examining how leadership practices operate under different organizational conditions and levels of institutional development.

### 3.3 Participants

Participants were selected through purposive sampling to ensure representation of key actors involved in leadership and organizational processes. The selection focused on individuals with direct experience in decision-making, teaching practices, and administrative functions (Table 2).

**Table 2:** Participant profile.

Category	Number	Role
**Principals**	3	Strategic leadership
**Teachers**	15	Instructional practice
**Staff**	6	Administrative support

Such sampling enables a comprehensive understanding of leadership practices from multiple organizational perspectives. Qualitative sampling prioritizes depth of information rather than statistical generalization (Patton, 2014).

### 3.4 Data collection methods

Data were collected through multiple qualitative methods to capture the complexity of leadership practices and organizational dynamics. The use of multiple sources strengthens data triangulation and enhances the credibility of findings (Lincoln and Guba, 1985) (Table 3).

**Table 3:** Data collection methods.

Method	Purpose	Output
**Interviews**	Explore perceptions and experiences	Narrative data
**Observation**	Capture everyday practices	Field notes
**Documents**	Examine institutional context	Policy and archival data

Semi-structured interviews were conducted to explore participants' perspectives on leadership practices and organizational processes. Observations focused on meetings, classroom interactions, and informal activities to capture leadership as it unfolds in practice. Document analysis included school policies, reports, and institutional records to provide contextual insights and support data triangulation.

### 3.5 Data analysis process

**Figure 2:**
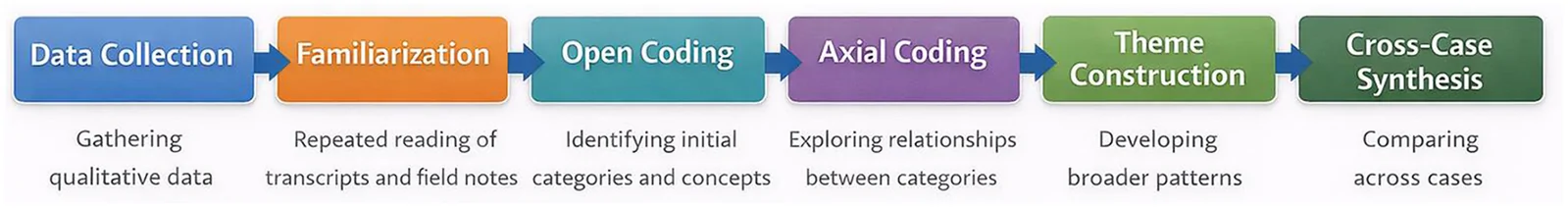
Thematic analysis process.

Data analysis followed a thematic analysis approach that allows for systematic identification, analysis, and interpretation of patterns within qualitative data (Braun and Clarke, 2006). The process was iterative and involved continuous movement between data and emerging interpretations (Figure 2).

Familiarization involved repeated reading of transcripts and field notes to develop an overall understanding of the data. Open coding was used to identify initial categories and concepts. Axial coding enabled the exploration of relationships between categories. Themes were then constructed to represent broader patterns related to work, agency, and professional culture. Cross-case synthesis facilitated comparison across the three schools, allowing identification of commonalities and contextual differences.

### 3.6 Trustworthiness and rigor

Several strategies were employed to ensure the rigor of the study. Credibility was enhanced through triangulation of data sources and methods. Member checking was conducted by sharing preliminary interpretations with participants to validate findings. Transferability was supported through rich, contextualized descriptions of the research setting. Dependability was ensured by maintaining a clear audit trail of data collection and analysis procedures. Confirmability was strengthened through reflexive practices to minimize researcher bias (Lincoln and Guba, 1985).

### 3.7 Ethical considerations

Ethical approval was obtained prior to the study. Participants were informed about the purpose of the research and provided voluntary consent. Confidentiality and anonymity were ensured by using pseudonyms and removing identifiable information. All data were securely stored and used exclusively for research purposes.

## 4 Findings

### 4.1 Transformative leadership in reconfiguring teachers' work

The findings show a clear shift in how teachers' work is organized and practiced across the three schools. Prior to leadership transformation, teachers' work was largely individual, routine-based, and focused on administrative compliance. Teaching, planning, and evaluation were conducted separately, with limited interaction among teachers. Professional roles were defined in fixed terms, leaving little room for adaptation or innovation.

A principal from MAN 3 described the earlier situation as follows:

“*Teachers mostly worked independently, and the main focus was completing administrative tasks.” (Principal, MAN 3)*

Teachers also reflected on the limited collaborative practices:

“*We prepared lesson plans individually, and there was not much discussion with colleagues.” (Teacher, MAN 3)*

“*Evaluation was more about documentation than improving teaching practices.” (Teacher, MAN 2)*

Leadership transformation introduced new forms of coordination and interaction, especially in MAN 1. Teachers began to engage in collaborative planning, shared reflection, and collective problem-solving. Work practices became more dynamic and responsive to classroom realities.

“*We now design lessons together and exchange ideas before teaching.” (Teacher, MAN 1)*

“*After class, we discuss what worked and what needs improvement.” (Teacher, MAN 1)*

*The principal emphasized the deliberate restructuring of work practices:*

“*We created regular spaces for collaboration so that teachers can learn from each other.” (Principal, MAN 1)*

Administrative staff also observed changes in coordination patterns:

“*Communication is more open, and teachers are actively involved in planning activities.” (Staff, MAN 1)*

*In MAN 2, elements of collaborative work have emerged, though not consistently across all teachers*.

“*Some teachers are already working in teams, while others still prefer to work individually.” (Principal, MAN 2)*

“*Collaboration happens in certain programs, but it is not yet part of everyday practice.” (Teacher, MAN 2)*

These findings are summarized in Table 4.

**Table 4:** Transformation of teachers' work.

Aspect	Before leadership change	After leadership change
**Teaching**	Individual	Collaborative
**Planning**	Routine-based	Innovation-driven
**Evaluation**	Administrative	Reflective
**Roles**	Fixed	Flexible

The transformation reflects a shift from *task-oriented work* toward *practice-oriented professionalism*. Teachers are no longer confined to isolated tasks. They participate in collective processes that shape instructional practices and organizational development.

### 4.2 Transformative leadership in expanding professional agency

Changes in work organization are closely linked to the expansion of professional agency. Agency refers to teachers' capacity to participate in decision-making, initiate innovation, and express professional perspectives within the school. The findings indicate that agency develops through leadership practices that encourage participation and dialogue.

In MAN 1, teachers described a strong sense of involvement in organizational processes.

“*We are invited to share our ideas before decisions are made.” (Teacher, MAN 1)*

“*Some of our ideas have been implemented as school programs.” (Teacher, MAN 1)*

The principal articulated this approach as part of leadership strategy:

“*Teachers are encouraged to contribute, not only to implement but also to shape school programs.” (Principal, MAN 1)*

Staff members also experienced increased participation:

“*We are included in discussions, especially when planning school activities.” (Staff, MAN 1)*

Agency is also visible in teachers' willingness to innovate in their professional practice.

“*We feel more confident to try new approaches in teaching.” (Teacher, MAN 1)*

“*There is support when we want to develop new learning methods.” (Teacher, MAN 1)*

In MAN 2, agency is present but varies among individuals.

“*Opportunities are available, but participation depends on each teacher.” (Principal, MAN 2)*

“*Some teachers take initiative, while others prefer to wait for direction.” (Teacher, MAN 2)*

In MAN 3, agency remains limited due to hierarchical structures and directive leadership.

“*Most decisions come from the leadership, and teachers follow the instructions.” (Principal, MAN 3)*

“*We usually carry out what has been decided, with little opportunity to give input.” (Teacher, MAN 3)*

The forms of agency identified in this study are presented in Table 5.

**Table 5:** Forms of professional agency.

Dimension	Manifestation	Example
**Decision-making**	Shared governance	Teacher forums
**Innovation**	Program creation	Digital learning
**Voice**	Open dialogue	Feedback systems

The findings indicate that agency is strengthened when leadership creates space for participation and recognizes teachers as active contributors. Teachers move from being implementers of policy toward becoming *co-creators* of organizational practices. This shift enhances professional engagement and supports continuous improvement within the school.

### 4.3 Transformative leadership in developing professional culture

The findings reveal that changes in work practices and professional agency contribute directly to the development of professional culture within the schools. Professional culture is not formed through formal policies alone. It emerges through repeated interactions, shared experiences, and collective engagement facilitated by leadership practices.

In MAN 1, leadership actively promotes dialogic communication and collaborative interaction. These practices create conditions in which shared norms gradually develop. Teachers described a shift in how they relate to colleagues and leadership.

“*We communicate more openly now, and discussions happen regularly, not only in formal meetings.” (Teacher, MAN 1)*

“*There is a sense that everyone can speak and be heard.” (Teacher, MAN 1)*

The principal highlighted the intentional effort to cultivate such a culture:

“*We encourage dialogue so that teachers feel comfortable sharing ideas and concerns.” (Principal, MAN 1)*

Staff members also observed the transformation of interaction patterns:

“*Coordination is based on discussion rather than instruction.” (Staff, MAN 1)*

These interactional changes support the emergence of shared norms, particularly related to collaboration, mutual support, and continuous improvement. Teachers no longer operate as isolated individuals. They function as members of a professional community.

“*We solve problems together, and that builds trust among us.” (Teacher, MAN 1)*

In MAN 2, elements of professional culture are developing, though unevenly across departments. Some groups demonstrate strong collaboration, while others maintain more traditional patterns.

“*There are teams that work very well together, but this is not yet consistent across the school.” (Principal, MAN 2)*

“*In some cases, we collaborate closely, but in others, communication is still limited.” (Teacher, MAN 2)*

In MAN 3, professional culture remains largely hierarchical. Communication flows from leadership to teachers, with limited opportunities for dialogue.

“*Most communication comes from the top, and discussions are quite limited.” (Teacher, MAN 3)*

“*We usually follow instructions rather than engage in collective decision-making.” (Staff, MAN 3)*

**Figure 3:**
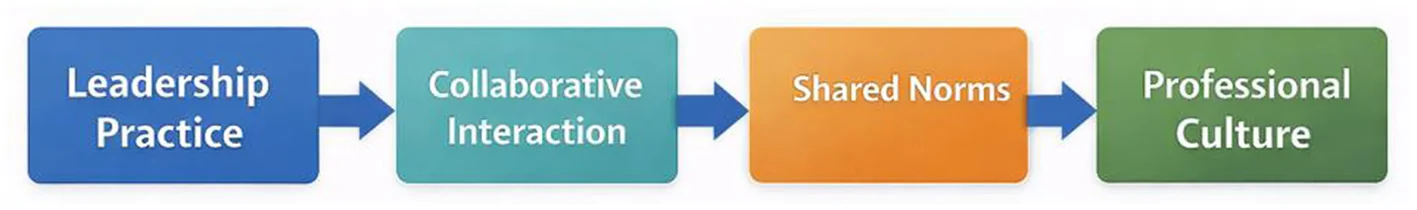
Model of professional culture transformation.

The transformation of professional culture is summarized in Figure 3 and Table 6.

**Table 6:** Cultural transformation.

Cultural element	Traditional	Transformative
**Communication**	Top-down	Dialogic
**Collaboration**	Limited	Intensive
**Innovation**	Rare	Continuous

This model illustrates how leadership practices initiate changes in interaction patterns. These interactions shape shared norms, which gradually form a professional culture.

The findings indicate that professional culture develops through sustained interaction rather than formal directives. Leadership influences this process by creating spaces for dialogue, encouraging collaboration, and supporting innovation. Culture becomes embedded in everyday practices and shapes how individuals engage with their work and with each other.

### 4.4 Organizational tensions

The transformation of work, agency, and culture is accompanied by several organizational tensions. These tensions reflect the complexity of implementing change within institutional structures that are shaped by regulation, tradition, and workload demands.

One major issue identified across all three schools is bureaucracy. Administrative requirements and policy compliance limit flexibility in implementing innovative practices.

“*There are many regulations that we must follow, and sometimes they restrict what we can do.” (Principal, MAN 2)*

“*We want to innovate, but administrative demands take a lot of time.” (Teacher, MAN 1)*

Staff members also emphasized the burden of administrative procedures:

“*A significant part of our work is focused on documentation and reporting.” (Staff, MAN 3)*

Another tension relates to resistance to change, particularly among senior teachers who are accustomed to established routines.

“*Some teachers are comfortable with existing practices and are hesitant to change.” (Principal, MAN 2)*

“*It takes time for everyone to adapt to new ways of working.” (Teacher, MAN 2)*

This resistance does not necessarily indicate rejection. It reflects differences in readiness and experience among teachers.

Workload also emerges as a significant constraint. The expansion of collaborative practices and innovation efforts increases the demands placed on teachers.

“*We are involved in many activities, and sometimes it feels overwhelming.” (Teacher, MAN 1)*

“*Balancing teaching, administration, and collaboration can be challenging.” (Teacher, MAN 2)*

These constraints are summarized in Table 7.

**Table 7:** Organizational constraints.

Issue	Impact	Example
**Bureaucracy**	Limits flexibility	Policy compliance
**Resistance**	Slows change	Senior teachers
**Workload**	Overburden	Administrative tasks

The findings show that organizational change is not linear. Leadership must navigate tensions between innovation and regulation, participation and hierarchy, as well as collaboration and workload. These tensions highlight the importance of adaptive leadership practices that can balance competing demands within the organization.

## 5 Cross-case analysis

The cross-case analysis identifies consistent patterns linking leadership orientation with variations in professional agency, organizational culture, and innovation across the three schools. Each case reflects a distinct configuration of leadership practice and its implications for the organization of work and professional interaction (Table 8).

**Table 8:** Comparison across schools.

Dimension	MAN 1	MAN 2	MAN 3
**Leadership**	Transformative	Hybrid	Administrative
**Agency**	High	Medium	Low
**Culture**	Collaborative	Emerging	Fragmented
**Innovation**	High	Moderate	Low

MAN 1 demonstrates a strong alignment between transformative leadership and organizational outcomes. Leadership is enacted through participatory practices, shared decision-making, and continuous interaction among school members. These practices create conditions that support high levels of professional agency. Teachers are actively involved in shaping programs and instructional strategies.

“*We involve teachers in discussions so they can contribute to decisions, not only implement them.” (Principal, MAN 1)*

Teachers describe their role as active contributors within the organization.

“*We feel responsible for the direction of the school because we are included in decision-making processes.” (Teacher, MAN 1)*

“*There is space to try new ideas, and support is provided when we implement them.” (Teacher, MAN 1)*

Staff members also experience inclusive organizational processes.

“*Communication flows through discussion, and coordination happens collectively.” (Staff, MAN 1)*

These interactional patterns support the development of a collaborative professional culture. Shared norms are formed through repeated engagement, creating trust and mutual responsibility. Innovation becomes embedded in everyday work practices rather than being limited to specific initiatives.

MAN 2 reflects a hybrid leadership orientation that combines participatory and hierarchical elements. This configuration produces varied outcomes across different organizational units. Opportunities for collaboration and participation exist, yet their implementation is uneven. Professional agency is present but differs among individuals and teams.

“*We provide opportunities for teachers to be involved, but engagement depends on each individual.” (Principal, MAN 2)*

Teachers describe differences in participation across groups.

“*Some teams work collaboratively, while others still rely on individual approaches.” (Teacher, MAN 2)*

“*There are opportunities to innovate, though not everyone takes them.” (Teacher, MAN 2)*

Staff observations indicate partial changes in organizational culture.

“*In some areas, communication is open, while in others it remains formal and structured.” (Staff, MAN 2)*

The professional culture in MAN 2 is developing but not yet fully institutionalized. Collaborative practices appear in specific contexts and are not consistently embedded across the organization. Innovation is moderate and often driven by particular individuals or teams rather than collective processes.

MAN 3 represents a predominantly administrative leadership model characterized by centralized decision-making and strong procedural control. Leadership emphasizes compliance and formal structure. This configuration limits opportunities for participation and constrains professional agency.

“*Most decisions are made by the leadership, and teachers follow established procedures.” (Principal, MAN 3)*

Teachers describe their work as structured and directive.

“*We focus on completing assigned tasks rather than discussing new ideas.” (Teacher, MAN 3) “Opportunities to contribute to decisions are very limited.” (Teacher, MAN 3)*

Staff members report similar patterns in organizational interaction.

“*Coordination is based on instructions, and discussions rarely occur.” (Staff, MAN 3)*

The professional culture in MAN 3 is fragmented, with limited collaboration and weak shared norms. Interaction follows formal lines of authority, reducing opportunities for collective learning. Innovation remains minimal due to restricted space for initiative and experimentation.

The comparison across cases demonstrates a clear relationship between leadership orientation and organizational outcomes. Transformative leadership is associated with strong professional agency, collaborative culture, and sustained innovation. Hybrid leadership produces mixed patterns, with partial development in agency and culture. Administrative leadership maintains stability while limiting adaptability and innovation.

These findings indicate that leadership functions as a mediating practice that shapes how work is organized and experienced. Leadership practices influence patterns of interaction, distribution of authority, and construction of professional roles. Differences across cases reflect variations in how leadership enables participation, trust, and shared responsibility.

Contextual conditions also shape the extent of transformation. Schools with existing collaborative practices show greater responsiveness to participatory leadership. Schools with rigid structures display slower adaptation and require gradual change processes. Leadership is embedded within institutional arrangements that define both opportunities and constraints.

The cross-case analysis highlights the relational and context-dependent nature of educational leadership. Leadership operates through practice rather than position, shaping organizational dynamics through interaction and coordination. Variation across cases provides empirical evidence that leadership plays a central role in reconfiguring work, enabling professional agency, and shaping professional culture within school organizations.

## 6 Conceptual model

**Figure 4:**
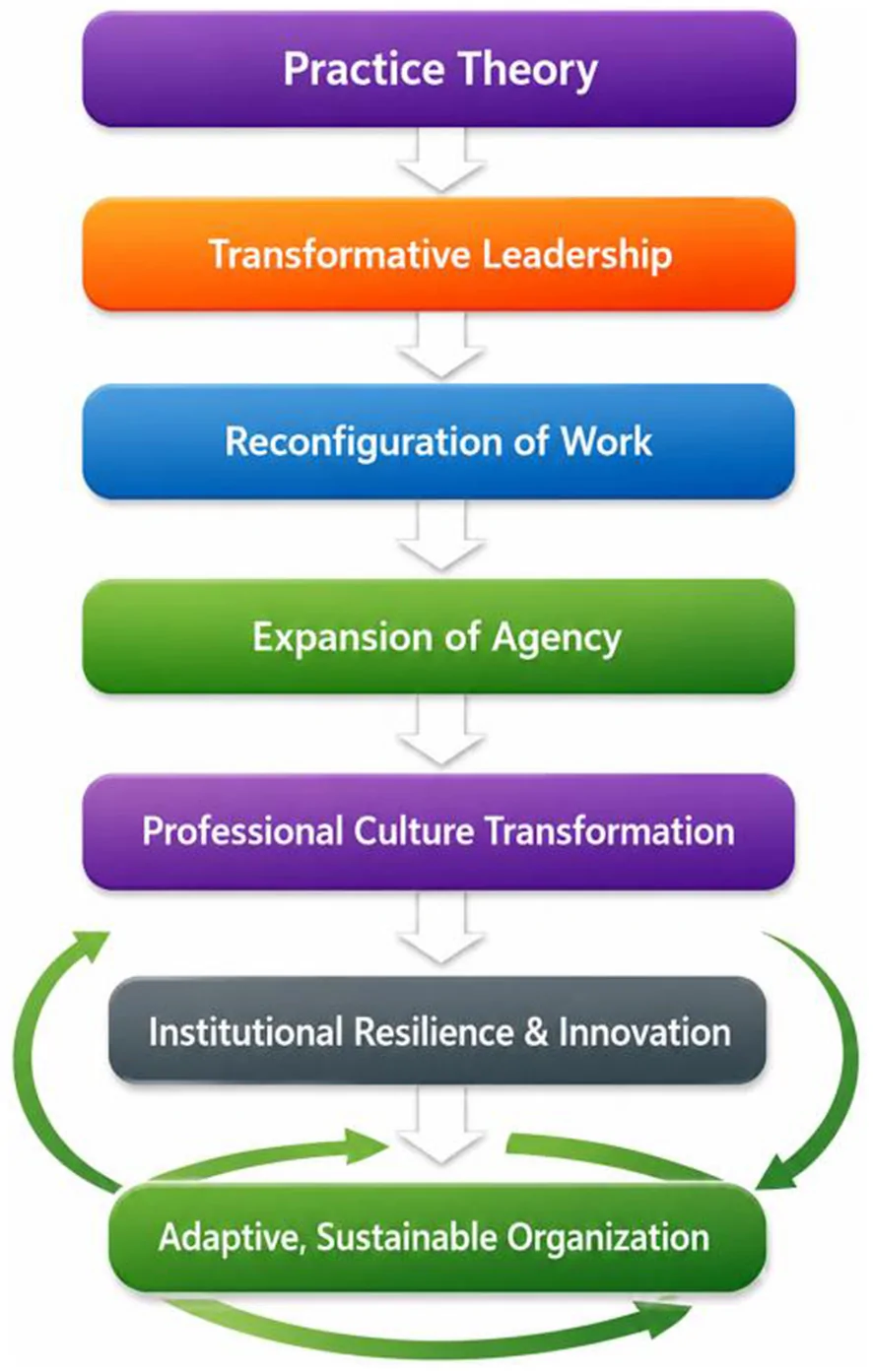
Transformative educational leadership model.

The conceptual model is derived from empirical findings that identify leadership as an organizational practice shaping the reconfiguration of work, the expansion of professional agency, and the transformation of professional culture within school settings. The model reflects a processual relationship grounded in everyday interactions observed across the three madrasahs (Figure 4).

Transformative educational leadership functions as the entry point of the model. Leadership is enacted through routine practices such as coordination, dialogue, and shared decision-making. Evidence from MAN 1 shows that leadership is embedded in collaborative planning sessions, reflective discussions, and collective problem-solving. Leadership is not limited to formal authority. It is produced through interaction that organizes institutional processes and professional relations.

Leadership practices restructure teachers' work. Findings indicate a shift from individual and routine-based labor toward collaborative and reflective professional activity. Teachers engage in joint lesson planning, peer evaluation, and continuous reflection. Professional roles become flexible and negotiated through interaction. Work is no longer defined by isolated tasks. It is constructed through collective engagement that connects instructional, administrative, and relational dimensions of practice.

The reconfiguration of work creates conditions for the expansion of professional agency. Teachers and staff gain opportunities to participate in decision-making processes and contribute to school development. In MAN 1, teachers describe involvement in program design and institutional planning. In MAN 2, participation appears uneven. In MAN 3, agency remains constrained by hierarchical structures. These variations indicate that agency emerges through interaction shaped by leadership practices and organizational context.

Professional culture develops through repeated interaction among organizational members. Findings show a transition from hierarchical and top-down communication toward dialogic and collaborative patterns. In MAN 1, communication is open and based on discussion. Shared norms of trust, mutual support, and collective responsibility become embedded in everyday practices. In MAN 3, culture remains fragmented and structured by formal authority. Cultural formation is linked to patterns of interaction rather than formal policy.

The interaction between leadership, work, agency, and culture produces organizational change. Schools with participatory leadership demonstrate stronger collaboration, higher levels of agency, and sustained innovation. Organizational change is expressed through adaptive practices, collective learning, and continuous improvement. The model captures how transformation emerges from practice rather than from formal directives.

The model also reflects structural constraints identified in the findings. Bureaucratic regulations, workload demands, and resistance to change shape the limits of transformation. Leadership mediates these conditions by interpreting policy requirements and creating space for localized adaptation. Transformation occurs within these boundaries and remains uneven across cases.

A recursive dynamic is present in the model. Professional culture and patterns of agency influence subsequent leadership practices. Interaction between structure, agency, and practice continues over time. Organizational change is not linear. It is produced through ongoing negotiation and coordination within a hybrid institutional context.

This conceptual model contributes to the sociology of work by positioning educational leadership as a mechanism that reorganizes labor processes, redistributes professional agency, and shapes workplace culture. Schools are understood as organizational arenas where work and social relations are continuously constructed through practice.

## 7 Discussion

### 7.1 Leadership as organizational practice

The findings reposition educational leadership as an organizational practice embedded in everyday interactions rather than a function tied to formal authority. Leadership is enacted through coordination, negotiation, and shared meaning-making processes that shape how work is structured within schools. This perspective aligns with practice-based approaches in organizational studies that view leadership as emerging from collective activity rather than individual control (Raelin, 2020).

Empirical evidence from MAN 1 illustrates how leadership operates through routine practices such as collaborative planning and reflective dialogue. These practices function as mechanisms of coordination that align individual efforts with organizational goals. Leadership is visible in how actors interact, distribute responsibilities, and construct shared understandings of professional work. This reinforces the argument that leadership is relational and processual, shaped through social engagement across organizational levels (Endrissat and von Arx, 2013).

The variation across cases demonstrates that leadership is context-dependent. Transformative leadership in MAN 1 generates participatory structures and shared responsibility. Hybrid leadership in MAN 2 produces partial engagement. Administrative leadership in MAN 3 maintains hierarchical control and limits interaction. These patterns indicate that leadership is embedded within institutional arrangements that influence how it is enacted. Organizational context defines the possibilities for leadership practice, including the extent of participation and collaboration (Thorpe et al., 2011).

### 7.2 Reconfiguring work in schools

The study shows that leadership practices contribute to a significant reconfiguration of teachers' work. Work shifts from individual and routine-based tasks toward collaborative and reflective professional practice. Teachers engage in joint planning, peer learning, and continuous evaluation. These practices reshape how work is experienced and organized.

This transformation reflects the emergence of collaborative professionalism, where teachers operate as members of a professional community rather than isolated individuals. Collaborative work practices create opportunities for shared learning and collective responsibility. Such developments are consistent with research highlighting the role of professional collaboration in improving educational outcomes and organizational learning (Vangrieken et al., 2015).

The findings also indicate that boundaries between formal and informal work become less rigid. Teachers participate in activities that extend beyond their assigned roles, including informal discussions, peer mentoring, and innovation initiatives. Work becomes fluid and adaptive, shaped by interaction rather than predefined tasks. This aligns with sociological perspectives that conceptualize professional work as dynamic and negotiated within organizational contexts (Evetts, 2011).

In schools where leadership remains administrative, work retains its rigid and task-oriented structure. Teachers focus on compliance and routine implementation. Opportunities for collaboration and reflection remain limited. The contrast across cases highlights the role of leadership in shaping not only what teachers do, but how they experience their work.

### 7.3 Agency and power dynamics

The expansion of professional agency observed in the findings reflects shifts in power relations within school organizations. Leadership practices that encourage participation create spaces where teachers can influence decisions and contribute to innovation. Agency emerges through interaction and is shaped by organizational conditions.

In MAN 1, teachers demonstrate high levels of agency, participating actively in decision-making and program development. This reflects a redistribution of power where authority is shared across organizational members. Leadership facilitates this process by enabling dialogue and recognizing teachers as contributors to organizational development. Such dynamics align with sociological perspectives that view agency as relational and embedded within social structures (Koudenburg et al., 2017).

Power remains structured by institutional frameworks. Bureaucratic regulations and accountability systems continue to define boundaries of action. Teachers operate within these constraints while navigating opportunities for participation. Leadership mediates this relationship by interpreting policies and adapting them to local contexts. This mediation enables a balance between control and autonomy.

In MAN 2, agency is uneven, reflecting variations in leadership practice and organizational culture. Some teachers engage actively, while others remain passive. In MAN 3, agency is limited due to centralized decision-making and hierarchical control. These differences illustrate that agency is not evenly distributed but depends on leadership practices and organizational conditions.

The findings suggest that leadership does not eliminate power asymmetry. It reshapes how power is exercised and distributed within the organization. Leadership creates conditions where agency can emerge, yet it remains embedded within structural constraints.

### 7.4 Contribution to sociology of work

This study contributes to the sociology of work by conceptualizing schools as complex organizational arenas where labor is continuously negotiated and reorganized. Teachers' work involves multiple dimensions, including instructional practice, collaboration, emotional engagement, and participation in organizational processes.

Leadership emerges as a central mechanism in the reorganization of labor. It shapes how work is distributed, how roles are defined, and how professional relationships are constructed. This perspective extends existing research by highlighting the role of leadership in structuring everyday work practices within educational organizations.

The findings also demonstrate that organizational change occurs through practice rather than formal policy alone. Change emerges through interaction, shared activity, and collective engagement. Leadership facilitates this process by creating spaces for collaboration and enabling new forms of work. This aligns with practice-based theories that emphasize the importance of routine activity in shaping institutional outcomes (Nicolini, 2012).

The study provides empirical insight from the Global South, where educational organizations operate within hybrid institutional contexts. Madrasah combine bureaucratic governance with cultural and religious expectations. Leadership plays a critical role in mediating these multiple logics, enabling schools to adapt while maintaining institutional coherence.

This contribution advances the sociology of work by integrating leadership, agency, and culture within a single analytical framework. It highlights how leadership functions as an organizational practice that reorganizes labor and shapes professional life. The findings underscore the importance of examining work as a relational and context-dependent process.

### 7.5 Hybrid professionalism and the sociology of professions in the global South

One of the most significant contributions of this study lies in its implications for the sociology of professions. The findings suggest that teachers' professional work within Indonesian madrasahs cannot be adequately explained through conventional distinctions between occupational professionalism and organizational professionalism (Evetts, 2011; Syafi'i et al., 2024; Abror et al., 2024). Teachers simultaneously experience bureaucratic accountability, collegial collaboration, religious obligations, and community expectations.

Evidence from MAN 1 demonstrates that transformative leadership expands opportunities for participation, collaboration, and professional discretion. However, these developments do not eliminate organizational accountability requirements. Instead, teachers continue to operate within formal regulatory structures while exercising greater professional agency. This indicates that professional authority is neither fully autonomous nor entirely controlled by organizational mechanisms.

The findings therefore support the emergence of what may be described as hybrid professionalism. Professional legitimacy is constructed through the interaction of multiple institutional logics, including state governance, professional collaboration, religious values, and community trust. Transformative educational leadership functions as a mediating mechanism that enables these diverse sources of authority to coexist within everyday organizational practice.

This perspective contributes to ongoing debates concerning professions in the Global South by challenging assumptions that professional development necessarily follows trajectories observed in Northern contexts. Rather than pursuing professional autonomy through jurisdictional closure, teachers in madrasahs appear to construct professional authority through collective responsibility, moral commitment, and institutional coordination. Consequently, professionalism in hybrid educational organizations may be better understood as a relational and contextually embedded achievement rather than as a fixed occupational status.

### 7.6 Leadership as jurisdictional mediation: a global south perspective

The findings extend existing sociology of professions scholarship by demonstrating that professional authority within Indonesian madrasahs is constituted differently from the trajectories commonly described in Northern professional systems (Evetts, 2009). Rather than seeking greater autonomy through jurisdictional closure, teachers operate within overlapping institutional domains involving bureaucratic accountability, pedagogical expertise, religious responsibility, and community expectations.

Transformative educational leadership functions as a mechanism of jurisdictional mediation through which these multiple sources of authority are coordinated and negotiated. Leadership does not simply empower teachers or reduce bureaucratic control. Instead, it reorganizes relationships among institutional actors and redistributes professional responsibilities across organizational, pedagogical, and religious domains.

Rather than reinforcing traditional views, this finding challenges historical assumptions that professional development inevitably leads to increasing occupational autonomy. Instead, it suggests that professional authority and autonomy are shaped by evolving institutional, organizational, and contextual conditions. In hybrid educational organizations of the Global South, professional legitimacy may derive less from exclusive jurisdiction and more from the capacity to coordinate multiple institutional logics (Battilana and Lee, 2014). Consequently, professionalism is better understood as a relational, negotiated, and contextually embedded achievement rather than a fixed professional status (Noordegraaf, 2015). The study therefore contributes not only to the sociology of work but also to the sociology of professions by proposing that leadership can be understood as a process of jurisdictional mediation within hybrid educational organizations.

### 7.7 Study limitations

Several limitations should be considered when interpreting the findings of this study. The investigation was conducted in three public Islamic senior high schools located within a single district in Indonesia. Although this setting offers substantial insight into leadership practices within hybrid educational organizations, the contextual specificity may limit the transferability of the findings to other educational environments.

The qualitative multiple-case study design emphasizes contextual richness and in-depth understanding rather than statistical representativeness. Consequently, the findings are intended to provide analytical insights that contribute to theory development and contextual interpretation rather than population-level generalizations.

Data collection relied primarily on participants' experiences, perceptions, and narratives, which were subsequently interpreted through the researchers' analytical lens. Despite the implementation of methodological strategies such as triangulation, member checking, and cross-case comparison to strengthen credibility and trustworthiness, the influence of subjective interpretation cannot be completely excluded.

The cultural, religious, and institutional characteristics embedded within the Indonesian madrasah system may also shape the enactment of leadership practices in ways that differ from other educational contexts. Further research conducted across diverse institutional settings, educational systems, and cultural environments would be valuable for examining the extent to which the identified patterns of leadership practice are transferable and theoretically applicable beyond the present context.

### 7.8 Future research directions

Future research may extend the present findings by empirically testing the conceptual framework through quantitative approaches, including structural equation modeling (SEM), to examine the relationships among transformative educational leadership, teachers' work, professional agency, and professional culture.

Comparative investigations across diverse educational sectors and national contexts may provide deeper insight into the ways institutional environments shape leadership practices and organizational transformation. Such studies would contribute to the identification of contextual factors that facilitate or constrain the development of transformative leadership within educational organizations.

Longitudinal research designs may further illuminate the dynamic nature of transformative leadership by examining how leadership practices develop over time and how organizational changes become embedded and sustained within school systems.

The integration of complementary theoretical perspectives, including Institutional Theory, Distributed Leadership Theory, Cultural-Historical Activity Theory (CHAT), and Organizational Learning Theory, may enrich the analytical understanding of the interrelationships among leadership, professional agency, and organizational culture. The incorporation of multiple theoretical lenses may also support the development of more comprehensive explanatory models of educational transformation.

Further scholarly attention may also be directed toward examining the long-term implications of transformative educational leadership for teacher well-being, organizational resilience, educational innovation, and sustainable school improvement. Such inquiry may contribute to a more comprehensive understanding of how leadership practices support enduring educational effectiveness and organizational sustainability in increasingly complex educational environments.

## 8 Conclusion

This study demonstrates that transformative educational leadership functions as an organizational practice that actively reorganizes labor within school settings. Leadership is not confined to formal authority or positional power. It is enacted through everyday interactions that coordinate work, shape professional relations, and structure organizational processes. The findings indicate a shift in teachers' work from individual, routine-based tasks toward collaborative and practice-oriented labor. Work becomes embedded in collective activities such as joint planning, reflective dialogue, and shared problem-solving. This transformation reflects a reconfiguration of the labor process, where professional roles are no longer fixed but negotiated through interaction. Professional agency expands within this context as teachers gain opportunities to participate in decision-making and contribute to organizational development. Agency emerges as a relational capacity shaped by leadership practices that enable dialogue, trust, and shared responsibility.

The study also reveals that organizational transformation is shaped by structural conditions that both enable and constrain professional action. Bureaucratic regulations, hierarchical traditions, and increasing workload pressures continue to define the boundaries of work and limit flexibility. Leadership operates as a mediating mechanism that interprets institutional demands and translates them into locally meaningful practices. This mediation creates space for agency while maintaining organizational coherence. The findings contribute to the sociology of work and organizations by positioning schools as complex labor arenas where work, power, and culture are continuously negotiated. The study advances theoretical understanding by integrating leadership, labor process, and agency within a unified analytical framework. Empirical evidence from the Global South highlights how organizational practices evolve within hybrid institutional contexts, offering insight into how leadership can reorganize work and reshape professional life while navigating structural constraints.

Beyond its contribution to Practice Theory and the sociology of work, this study contributes to the sociology of professions by demonstrating that professionalism within Indonesian madrasahs cannot be reduced to either occupational professionalism or organizational professionalism. Instead, professional authority emerges through the interaction of bureaucratic, pedagogical, religious, and community-based expectations. The findings therefore support the concept of hybrid professionalism as a useful framework for understanding professional work in educational organizations across the Global South.

This study contributes to the sociology of professions by illustrating the usefulness of Evetts (2011) distinction between occupational and organizational professionalism through a case of hybrid professionalism in an Indonesian Islamic educational context (Evetts, 2011). The findings suggest that, within this particular context, professional jurisdiction is relational and shared rather than exclusively controlled by professional actors. They further indicate that transformative educational leadership can function as a mechanism of jurisdictional mediation by coordinating bureaucratic, pedagogical, and religious domains of professional practice. While these findings offer insights into the dynamics of professional authority in Global South educational settings, their applicability should be understood within the specific institutional and regional context examined in this study.

Theoretically, this study extends Practice Theory into the field of educational leadership by demonstrating how leadership practices function as mechanisms for reorganizing work, enabling professional agency, and transforming professional culture. Unlike conventional leadership studies that emphasize individual leaders, this research conceptualizes leadership as an organizational practice emerging through collective interaction. This contribution offers a sociological explanation of educational change within hybrid institutional contexts of the Global South.

## Data Availability

The raw data supporting the conclusions of this article will be made available by the authors, without undue reservation.
